# A Glimpse Into the Sexual Dimorphisms in Major Depressive Disorder Through Epigenetic Studies

**DOI:** 10.3389/fncir.2021.768571

**Published:** 2021-10-20

**Authors:** Branden Cahill, Samuel Poelker-Wells, Jonathan F. Prather, Yun Li

**Affiliations:** Department of Zoology and Physiology, University of Wyoming, Laramie, WY, United States

**Keywords:** social isolation, early life stress, social defeat, single-cell RNA sequencing, stress, rodent models, sexual dimorphism, epigenetics

## Abstract

Depression is an umbrella term used to describe a mood disorder with a broad spectrum of symptoms including a persistent feeling of sadness, loss of interest, and deficits in social behavior. Epigenetic research bridges the environmental and genetic landscape and has the potential to exponentially improve our understanding of such a complex disorder. Depression is also a sexually dimorphic disorder and variations exist within epigenetic modification sites between sexes. These sex-specific mediators may impact behavioral symptomology and could serve as therapeutic targets for treatments to improve behavioral deficits. This mini review will focus on the social behavior perspective of depression and specifically explore the sexually different epigenetic modifications on depression.

## Introduction

Depression, also referred to as major depressive disorder (MDD), or major depression, is characterized by a range of clinical symptoms based on DSM-V criteria. In order to be diagnosed with MDD, patients must experience a depressed mood that lasts at least two weeks. Depressive manifestations include feelings of low self-esteem and a loss of interest or pleasure in activities that the patient previously enjoyed. Behaviorally, depression can be evident as reduced vigor, circadian dysfunction, eating disorders, or difficulty concentrating on important tasks. These challenges can constitute a condition that has devastating impacts directly for the patient and indirectly for others who have close relationships with the patient.

Impaired social functioning is a hallmark of depression and is correlated with the severity of depression (Hirschfeld et al., 2000; Rhebergen et al., 2010). Major depressive disorder patients tend to spend less time interacting socially and develop less depth within friendships than healthy individuals (Elmer and Stadtfeld, 2020). Additionally, social isolation and social deficits can contribute to the emergence of depressive symptomology (Jose and Lim, 2014). Consistent with this relationship, patients’ reporting of feelings of loneliness is strongly associated with behavioral symptoms of depression during social isolation in young adults (Matthews et al., 2016).

The global rise in the number of MDD diagnoses points to the need for effective therapies. Over the past 50 years, many new therapies have emerged, such as pharmacological approaches that have yielded 5 classes of pharmacological antidepressants consisting of selective serotonin reuptake inhibitors (SSRIs), serotonin and norepinephrine reuptake inhibitors (SNRIs), tricyclic antidepressants (TCAs), monoamine oxidase inhibitors (MAOIs) and atypical antidepressants drugs. These pharmacological treatments have clinical benefit (Hillhouse and Porter, 2015), but are therapeutically effective in less than 50% of patients and this have not improved significantly over time (Berton and Nestler, 2006). It is estimated that 10–30% of MDD patients completely fail to respond to these treatments which is considered treatment-resistant MDD (Rush et al., 2006; Conway et al., 2017). These broad therapeutic strategies also have a latency to provide benefit and can cause dire side effects that lead patients to withdraw from treatments (Fava, 2000; Uher et al., 2011; Cartwright et al., 2016; Wang et al., 2018; Sobieraj et al., 2019).

Notably, therapeutic effects vary greatly between the sexes. For example, SSRIs, SNRIs and other pharmacological antidepressants such as mood stabilizers, have a significantly greater effects on women compared to men (Kornstein et al., 2000; Khan et al., 2005; Berlanga and Flores-Ramos, 2006; Seney and Sibille, 2014; Charlotte et al., 2015). These sex-based differences highlight the necessity to understand the sex differences in molecular and circuit mechanisms of depression. In this mini review we summarize recent progress in epigenetic research revealing epigenetic target sites corresponding to depressive behaviors. Specifically, we focus on the sexually dimorphic epigenetic factors controlling social deficits involved in MDD.

## Sex, Social Behavior and Depression, Where Is the Bridge?

For about 50 years, it has been consistently reported that women are diagnosed with MDD at almost 2-fold greater rate than men (Weissman and Klerman, 1977; Ford and Erlinger, 2004). Yet, men who suffer with depression have a 10-fold greater rate of suicide attempts than women (Blair-West et al., 1999). Many psychosocial theories are proffered as to why sexual dimorphisms exist in the diagnosis of depression (Jorm, 1987; Mehl-Madrona et al., 2019). However, such stark sexual differences in depression also highlight the need to understand the neurophysiological differences between the sexes.

There are many baseline differences between female and male social behaviors (Eagly and Steffen, 1986; Halpern et al., 2007; Gur et al., 2012). Hormonal and chemosensory signals are integrated in specific brain regions that control social behavior (Newman, 1999; Rolls, 2004; Amodio and Frith, 2006). Neuroimaging studies on human neurology have suggested these behavioral differences may be influenced by sex differences in neuroanatomy and structural connectome (Gur et al., 1999; Goldstein et al., 2001; Cosgrove et al., 2007; Ingalhalikar et al., 2014). A recent study using single-cell RNA sequencing of the mouse ventrolateral subdivision of the ventromedial hypothalamus, revealed some transcriptomic types exhibiting differential expressions in males and females, providing the first evidence of the existence of sex specific neurons in the mammalian CNS (Kim et al., 2019). In addition, it has also been suggested that genetic, epigenetic and environmental factors all have impacts on sex differences in social behavior (Manuck et al., 2000; Hammock and Young, 2004; Shepard et al., 2009; Aspe-Sanchez et al., 2015; Dumais and Veenema, 2016).

Epigenetics refers to changes in gene expression in the absence of alterations of the genome sequence (McCarthy et al., 2009). Primary mechanisms of epigenetic regulation include DNA methylation and demethylation, histone modifications, and non-coding RNAs known as microRNAs [for reviews, please see (Peters and Schubeler, 2005; Klose and Zhang, 2007; Li et al., 2007; Bannister and Kouzarides, 2011; Ha and Kim, 2014)]. These mechanisms are important for regulations of transcription profiles and non-coding RNA expression, whose disruptions have significant impacts on cellular functions and therefore lead to diseases (Portela and Esteller, 2010). Recent breakthroughs demonstrated that stress-induced epigenetic modifications not only have been implicated in the development of MDD (van der Doelen et al., 2014; Jawahar et al., 2015), but have also been reported to be transmitted across generations (Franklin et al., 2010; Dietz et al., 2011; Gapp et al., 2014, 2020; Short et al., 2016; Pang et al., 2017; Jawaid et al., 2018; Cunningham et al., 2021).

## Epigenetic Regulation and Sex Differences in Animal Models of Major Depressive Disorder Relevant to Social Behavior

Studies in rodent models of depression have revealed that specific depressive behaviors relate to specific dysfunctions of neural mechanisms (Yoon et al., 2014). It is also suggested that different causes of one’s depression, such as different early life traumas, could prompt variations of neural mechanistic issues (Pacak and Palkovits, 2001; Goldstein, 2010; Smith and Pollak, 2020). In light of these many possible mechanistic variations, it is not surprising that manifestations of depression can vary greatly between individuals. If we are to achieve a complete understanding of the mechanisms that underlie MDD, it will be necessary to consider not only epigenetic mechanisms, but also how they are modulated in different ways in different social contexts. To perform those investigations, researchers have developed a variety of animal models of depression. Table 1 lists comparisons of studies on a variety of rodent models of depression. Here we focus on epigenetics studies in rodent models either exhibiting social deficits or implementing social stressors.

**TABLE 1:** Comparisons of most epigenetics studies on rodent models and human MDD.

Model	Depression Expression and which test used	Social deficits Expressed	Sex Differences Found	If epigenetic influence found- single nuclei or homogenous analysis and which region(s)	References
(A) Chronic Social Defeat	1- Sociability 2- Sociability, SP, TS, EPM, AD 4- Sociability, EPM, FST 5- Sociability, OFT, EPM, FST, TS 6- Sociability 7- AD, FST 8- Sociability 9- Sociability, AD 10- Sociability, weight, food intake	1, 2, 4, 5, 6, 8, 9, 10,	2, 3, 6	1- homogenous (hippocampus and PFC) 3- single nuclei (ventromedial hypothalamus) 4-homogenous (PFC) 5-homogeous (NAc) 7- homogenous (NAc) 8- homogenous (genes) 9- homogenous (hippocampus) 10- homogenous (blood, hippocampus)	(1) Mallei et al., 2019 (2) Iniguez et al., 2018 (3) Kim et al., 2019 (4) Reshetnikov et al., 2021 (5) Qian et al., 2020 (6) Lin et al., 2021 (7) LaPlant et al., 2010 (8) Elliott et al., 2010 (9) Tsankova et al., 2006 (10) Razzoli et al., 2011
(B) Chronic Variable/Mild/Unpredictable Stress	1- OFT, EPM, IA 2- ST, NSF, SP, FST, EPM, Locomotion, CORT 3- FSR 4- CORT, weight 5- FST, OFT, AD 6- AD 7- CORT, OFT, SP 8- SP, EPM, FST, OFT 9- OFT, FST 10- OFT, SP, CORT, AD 11- AD, SP	1, 4,	2, 3, 5, 6, 7, 8, 9, 10, 11	1-homogenous (hippocampus) 2- single-nuclei (NAc) 3- homogenous (NAc) 4- homogenous (hippocampus, PFC, paraventricular nucleus (PVN) 10- homogenous (hippocampus) 11- homogenous (orbitofrontal coretx, hippocampus)	(1) Viana Borges et al., 2019 (2) Hodes et al., 2015 (3) LaPlant et al., 2009 (4) Witzmann et al., 2012 (5) Rincon-Cortes and Grace, 2017 (6) Elakovic et al., 2011 (7) Lu et al., 2015 (8) Sachs et al., 2014 (9) Shepard et al., 2016 (10) Xing et al., 2013 (11) Pitychoutis et al., 2012
(C) Early Life Stress	1- Sociability, OFT, FST 2- Addiction 3- OFT, SP, EPM, FST 4- FST 5- OFT, EPM, FST, CFP 7- OFT, FST, AD 8- FST, OFT, EPM, CORT	1, 2, 5, 6, 7,	1, 2, 3, 5, 6, 8	1- homogenous (NAc) 2-homogenous (PFC, NAc, ventral tegmental area, mA) 3-homogenous (NAc) 4-homogenous (hippocampus) 5- homogenous (hippocampus) 6-single-nuclei (striatum, NAc, amygdala, hippocampus) 7-homogenous (ventral hippocampus)	(1) Kronman et al., 2021 (2) Walker et al., 2021 (3) Lei et al., 2020 (4) Seo et al., 2020 (5) Sun et al., 2021 (6) Catale et al., 2020 (7) Chang et al., 2020 (8) Brummelte et al., 2012
(D) Flinder Line	1- FST, AD	1		1-single nuclei (hippocampus and PFC)	(1) Marchetti et al., 2020
(E) Human MDD	1- Suicide, proxy-based interviews 2- GSK-HiTDiP 3- MADRS-S, DAWBA depression band, SUAS 4- ST, NSF, SP, FST, OFT, EPM 5- PSS, AD 6- Suicide 7- ST, NSF, SP, FST, OFT, EPM 8- ACE 9- CECA, Suicide 10- Suicide 11- Suicide 12- Suicide 13- DNHS 14- Suicide, proxy-based interviews 15- Suicide, proxy-based interviews 16- Suicide, CECA 17- Suicide, proxy-based interviews 18- HAM-D 19- Suicide 20- Suicide, MDD 21- Suicide, MDD	4, 8	7	1-single nuclei (dorsolateral PFC) 2-homogenous (blood) 3- homogenous (hippocampus) 4-single nuclei (mPFC, NAc) 5-homogenous (saliva) 6-homogenous (FCx and hippocampus) 7-homogenous (ventromedial PFC, dorsolateral PFC, anterior insula, NAc, ventral subiculum) 8- homogenous (sperm/egg) 9- homogenous (hippocmpus) 10- homogenous (hippocampus) 11- homogenous (orbitoPFC, subgenual cingulate gyrus) 12- homogenous (PFC) 13- homogenous (blood) 14- homogenous (PFC) 15- homogenous (hippocampus, GR) 16- homogenous (hippocampus, GR, cingulate cortex) 17- homogenous (hippocampus, PFC, blood) 18- homogenous (blood) 19- homogenous (middle temporal gyrus) 20- homogenous (orbital frontal cortex) 21- homogenous (frontal cortex, cerebellum)	(1) Nagy et al., 2020 (2) Leday et al., 2018 (3) Ciuculete et al., 2020 (4) Scarpa et al., 2020 (5) Palma-Gudiel et al., 2021 (6) (Misztak et al., 2020) (7) Labonte et al., 2017 (8) Dickson et al., 2018 (9) Labonte et al., 2012a (10) Labonte et al., 2013 (11) Murthy and Gould, 2018 (12) Schneider et al., 2015 (13) Uddin et al., 2010 (14) Nagy et al., 2015 (15) McGowan et al., 2008 (16) Labonte et al., 2012b (17) Chen et al., 2011 (18) Lopez et al., 2013 (19) Aston et al., 2005 (20) Ernst et al., 2009a (21) Ernst et al., 2009b
	22- Suicide, MDD, CECA 23- Suicide. MDD 24- Suicide			22-homogenous (cerebellum, hippocampus) 23- homogenous (PFC) 24- homogenous (frontopolar cortex, amygdala, PVN)	(22) McGowan et al., 2008 (23) Fiori and Turecki, 2010 (24) Poulter et al., 2008

*Forced Swim Test = FST; Sucrose Preference = SP; Tail Suspension = TS; Restraint Stress = RS; Splash Test = ST; Novelty suppressed feeding = NSF; Open Field Test = OFT; Elevated Plus Maze = EPM; Anti-depressant treatment = AD; Inhibitory Avoidance = IA; Contextual Fear Paradigm = CFP; ACE questionnaire = ACE; Perceived Stress Scale = PSS; Suicide Assessment Scale = SUAS; Montgomery-Åsberg Depression Rating Scale-Self = MADRS-S; Development and Well-Being Assessment = DAWBA; GlaxoSmithKline-High-Throughput Disease-specific target Identification Program = GSK-HiTDiP; Cortisol levels = CORT; Childhood Experience of Care and Abuse interviews = CECA; Detroit Neighborhood Health Study = DNHS; Hamilton Rating Scale of Depression = HAM-D; Professional MDD diagnosis = MDD.

### Chronic Social Defeat Stress Model

The Chronic Social Defeat Stress (CSDS) paradigm induces a range of depression-like behaviors in male mice including social withdrawal, anxiety, helplessness, anhedonia, memory deficits and decreased locomotion (Avitsur et al., 2001; Planchez et al., 2019). Several observations emerged with epigenetic studies in the CSDS paradigm. A selective reduction of brain derived neurotrophic factor (BDNF-6) transcript in the hippocampus and an increase of BDNF-4 transcript in the prefrontal cortex (PFC) were found in susceptible males (Mallei et al., 2019). Moreover, enzymes important for epigenetic modifications were also changed in susceptible males. For example, g9a mRNA was reduced in the hippocampus (Mallei et al., 2019); HDAC5 and DNMT3a mRNA levels were reduced in the PFC (Mallei et al., 2019); expression of HDAC7 was reduced in the nucleus accumbens (NAc) (Qian et al., 2020). Thus, the CSDS model affords an opportunity to discern the relationship between specific epigenetic mechanisms and the emergence and progression of depressive phenotypes.

One shortcoming of the CSDS model is the difficulty to find a suitable intimidator to implement CSDS on female mice, because of their generally docile behavior toward one another (Beery and Zucker, 2011). Additionally, females are more susceptible to develop depression-like behaviors from psychosocial stress than physical intimidation (Haller et al., 1999; Kessler, 2003). Recently, a paradigm known as vicarious chronic social defeat stress (vCSDS) was developed to induce depression-like behaviors solely using psychological stress. It was reported that vCSDS triggered significant decreases in sociability, bodyweight and sucrose preference, increased helplessness and higher levels of blood corticosterone in both male and female mice compared to controls (Warren et al., 2013; Iniguez et al., 2018). Interestingly, there were also sexual dimorphisms in response to vCSDS such that no significant anxiolytic response was induced in females yet was evident in males (Warren et al., 2013; Iniguez et al., 2018). Therefore, vCSDS model provides a practical way for researchers to investigate sex differences in epigenetic modifications associated with the adverse social conditions.

### Chronic Variable Stress Model

Chronic variable stress (CVS) is a paradigm commonly used to induce long-term stress related mood disorders including depression (Cotella et al., 2019). Chronic variable stress procedure can lead to decreases in appetite, abnormalities in circadian rhythm cycle, elevations in corticosterone and adrenal levels along with decreases in sucrose preference (Herzog et al., 2009; Planchez et al., 2019). This paradigm uses a multitude of stress-inducing methods over time to reveal stress vulnerabilities in male and female rodents (Strekalova et al., 2011). It seems easier for the CVS paradigm to induce behavioral changes from females than males (Borrow et al., 2018). Other variations of the CVS model include the chronic mild stress model (CMS) (Willner, 2017), as well as unpredictable variable stress models (Kessler, 1997; Kendler et al., 1999).

In general, social deficits are hard to recapitulate within CVS paradigms without the combination of social stressors (Witzmann et al., 2012; Viana Borges et al., 2019). Some researchers have adopted the use of combinatorial stress paradigms known as chronic social instability within CVS (Haller et al., 1999; Goni-Balentziaga et al., 2018). A recent epigenetic study using a combination of social isolation and unpredictable CMS revealed significantly increased HDAC5 expression, decreased H3K9 and H4K12 acetylation, and reduced BDNF levels in the hippocampus leading to impaired long-term memory (Viana Borges et al., 2019). Nevertheless, CVS model alone is a good tool to study sex differences in epigenetic modifications corresponding to depression-like behaviors other than social deficits.

### Ealy Life Stress Model

Ealy Life Stress (ELS) model attempts to replicate traumatic stress from early life experiences (Murthy and Gould, 2018). Early life adversity may induce drastic and long-lasting epigenetic modifications in key regulatory genes of stress response (Nusslock and Miller, 2016; Nelson et al., 2017). A typically used ELS model is the postnatal maternal separation paradigm (Newport et al., 2002; Millstein and Holmes, 2007; Anier et al., 2014). Maternal separation can induce gene transcription changes during neurological development, leading to disturbances in cognition, learning, and emotion (Vranceanu et al., 2007; Tyrka et al., 2009). Moreover, maternal separation also has long-term effects into adulthood, such as increased susceptibility to stress, anxiety, depression and impaired spatial navigation learning (Gross et al., 2012). A study focusing on the long-term effects from maternal separation found that young adult and middle-aged mice exhibited decreases in glucocorticoid receptor (GR) expression, increases in HDAC5 levels and decreased histone acetylation in the hippocampus. The extent of these changes were greater in middle-aged mice than young adult mice, indicating that these epigenetic changes are long-lasting (Seo et al., 2020). Rodents subjected to more intense maternal separation exhibited more significant behavior changes in anxiety, depression and contextual fear memory, correlated with more diminished BDNF mRNA and protein levels, decreased H3K9 acetylation and increased HDAC2 levels in the hippocampus (Sun et al., 2021). Mice subjected to postnatal maternal separation and then CSDS in adulthood displayed increased susceptibility to CSDS, along with trimethylation of the 4th lysine residue of histone H3 (H3K4me3) in the PFC (Reshetnikov et al., 2021), and dimethylation of lysine 79 of histone H3 (H3K79me2) in the NAc (Kronman et al., 2021).

Other paradigms used in the ELS model include early social isolation (ESI) paradigm- where each pup is singly housed, and the early social stress paradigm (ESS) paradigm- where each pup is housed with an adult male CD-1 aggressor mouse. Comparisons of ESI and ESS on epigenetic reprogramming concluded that different stressful early life experiences engendered different impacts on DNA methylation levels in specific brain regions. Particularly, ESI induced more drastic effects on DNA methyltransferases and caused significantly reduced expression levels of Dnmt1, Dnmt3a, and Dnmt3b (Catale et al., 2020). Besides DNA methylation and histone modification, microRNAs also contribute to epigenetic modifications in ELS model. For example, overexpression of microRNA-206 in sensory neurons reduces BDNF expression in cell bodies and axons (Shrestha et al., 2019). Another study utilizing ESI in male mice found that ESI susceptible mice had elevated levels of microRNA-206 and reduced BDNF mRNA in the ventral hippocampus compared to controls (Chang et al., 2020).

Recently, it was found that prenatal stress on offspring triggered more display of anxiety-like behaviors in females and more depression-like behaviors in males. These behavioral differences are correlated with sexually different methylation patterns on the promotor region of GR genes, levels of DNA methyltransferases (Dnmt1 and Dnmt3a), and DNA demethylase (Tet methylcytosine dioxygenase 2) (Lei et al., 2020). In general, ELS models recapitulate social deficits induced by early life adversity and are good models to investigate sex differences in epigenetic modifications in MDD.

## Epigenetic Regulation and Sex Differences in Human Studies of Major Depressive Disorder

Studies in epigenetic modifications of human MDD have recently begun to grow, but investigations into sex differences are still rare. Recent studies have found sexually different methylation patterns in the dorsolateral PFC of late-life depression (Huls et al., 2020). Ealy life stress studies have revealed sex differences in methylation changes at the promoter of *NR3C1* and the regulatory region of the *FKBP5* locus (Hill et al., 2019; Wiechmann et al., 2019). Another study discovered a significant sex difference in methylation of the promoter of oxytocin gene in MDD patients (Sanwald et al., 2020). Transcriptional studies have also shown sex-specific transcriptional signatures in human MDD which might be due to epigenetic modifications. For example, Labonte et al. (2017) explored the differential expression and weighted gene coexpression network analyses between male and female MDD patients across the ventromedial and dorsolateral PFC, the anterior insula, NAc, and the ventral subiculum then compared the results with CVS mouse profiles (Labonte et al., 2017). They were able to identify sex-specific gene coexpression modules significantly associated with MDD and hub genes that carry important functional roles such as *DUSP6* downregulation in females and *EMX1* overexpression in males (Labonte et al., 2017).

MDD may arise in part from the differential expressions and actions of the same gene across brain regions. Ciuculete et al. (2020) discovered higher methylation levels within the hepatocyte growth factor receptor (*MET*) gene associated with higher depression scores and susceptibility for suicidal symptoms, along with an inverse relationship to mRNA levels of both hepatocyte growth factor (HGF) expression and MET expression in the hippocampus (Ciuculete et al., 2020). Misztak et al. (2020) discovered significant decreases in H3K9/14ac expression, BDNF protein levels and p-S421-MeCP2/MeCP2 protein ratio in both the frontal cortex (FCx) and hippocampus, along with significant increases in HDAC3 protein levels and H3K27me2 expression in both the FCx and hippocampus, as well as increases in Sin3a in the hippocampus in suicide victims (Misztak et al., 2020). This suggests that the lowered BDNF protein levels in suicide victims were most likely due to decreases in histone acetylation and increased levels of factors related to deacetylation and methylation along with MeCP2 factor which may act bidirectionally (Misztak et al., 2020).

Importantly, systematic comparisons in transcriptional profiles between human MDD and three different mouse models, including CVS, social isolation and CSDS, observed the shared transcriptional signatures between human and mouse models in two brain regions, the medial PFC and NAc (Scarpa et al., 2020). Specifically, CVS and social isolation each replicated ∼20% of the transcriptional changes in humans MDD in the PFC and NAc whereas, CSDS recapitulated ∼4% changes in gene expression. These results not only reveal significant overlaps in human MDD and mouse models, but also highlight different mouse models recapturing distinct aspects of human MDD.

Interestingly, a recent study investigating transgenerational epigenetic changes of depressive disorders associated reductions in microRNA-449 and microRNA-34 in sperm of both men and mice exposed to chronic ESI (Dickson et al., 2018). These microRNA deficits persisting in sperm promoted anxiety and social deficits in their offspring across generations (Dickson et al., 2018). This study supports the notion that epigenetic mechanism controls gene expression in a heritable way.

Most transcriptional and epigenic studies in human MDD use bulk homogenates of tissues, concealing potentially distinct changes in gene expression from individual cell types. Advanced examination using single-nucleus RNA-sequencing (snRNA-seq) on transcriptomics of the dorsolateral PFC in MDD patients, identified 26 cellular clusters of which, 60% revealed differential gene expression from controls, with the greatest dysregulation found in deep layer excitatory neurons and immature oligodendrocyte precursor cells associated with altered expression of *PRNP* and *KAZN* genes (Nagy et al., 2020). These results highlight the importance of exploring cell-type specific mechanisms in the development of MDD.

## Conclusion and Future Directions

It is evident that current pharmacological treatments cause widespread/non-specific side effects in many MDD patients, and patient testimonials suggest these effects do not bring them back to a baseline of normalcy or bring stability of mind (Wang et al., 2018). Limited efficacy and tremendous side effects of existing treatments should compel the scientific community to develop better targeted and individualized interventions.

Two obstacles in exploring pathogenic mechanisms of MDD are the extremely broad clinical manifestations and the unique individual etiological triggers. Epigenetics is an exciting approach to bridge environments, genes and behaviors, as it investigates environmental influences on gene expressions that may produce individual and sex-based discrepancies in behavior (West and Greenberg, 2011).

Human epigenetic studies are rapidly expanding. However, most studies explore general epigenetic abnormalities in MDD, lacking the sexual and behavioral specificity. Various rodent models provide powerful tools to ameliorate specific aspects of different depression-like phenotypes (Catale et al., 2020; Chang et al., 2020; Lei et al., 2020; Kronman et al., 2021). For example, social deficits in MDD are easily and superiorly recapitulated in the ELS models with maternal separation and social isolation paradigms. ELS, as well as, CSDS/vCSDS models, have great potential to reveal sex differences within the epigenetic and behavioral responses to stress.

Major depressive disorder (MDD) research has only begun transitioning to explore the underlying mechanisms for evident sexual dimorphisms in MDD. With the rapid maturation and increasing commercial availability for new techniques such as snRNA-seq, future research hopefully will reveal epigenetic machinery toward accurate understanding of the sexual dimorphic and specific aspects of behavioral deficits in MDD.

This table compiles results from most epigenetics studies on rodent models and human MDD. In this table, we endeavor to show the progress seen in recent decades from early research that only included male subjects transitioning into exploring the sexually different epigenetic modifications. **(A)** Chronic social defeat stress and vicarious chronic social defeat stress is a model in which subjected rodents are repeatedly physically defeated by a larger, more aggressive strain or repeatedly view the physical defeat of another rodent of the same strain via a larger, more aggressive strain for psychosocial stress. **(B)** The chronic variable stress model utilizes a variety of stressors in order to stress subjects in order to express depressive phenotypes. Given the selection of stressors, researchers attempt to express specific depressive phenotypes such as social deficits. Articles utilizing social stressors are in blue. **(C)** The early life stress model has been the most popular model for MDD from our findings. The early life stress model administers one of various stressors, the most common being maternal separation before weaning is supposed to occur. Some of these stressors can be administered to the dam before the birth of her litter. This model has provided the most robust depressive phenotypes as of recent and can reliably express the social deficit phenotypes when social paradigms are used that are seen in MDD. **(D)** The FSL model is a line of rodents that physiologically express many neural dysfunctions as well as some behavioral dysfunctions seen in MDD like decreased serotonin synthesis, reduced BDNF expression and anxious social interactions. **(E)** Human MDD study utilizes the post-mortem tissues of MDD patients. Most cases do not explore the specific stress phenotypes that patients experienced because testimonial accounts are limited and thus, it is hard to study MDD associated stressful behaviors associated with mechanistic and genetic influences.

## Author Contributions

BC, JP, and YL contributed to the preparation of the manuscript. BC and YL wrote the manuscript. JP, SP-W, and YL edited the manuscript. All authors contributed to the article and approved the submitted version.

## Conflict of Interest

The authors declare that the research was conducted in the absence of any commercial or financial relationships that could be construed as a potential conflict of interest.

## Publisher’s Note

All claims expressed in this article are solely those of the authors and do not necessarily represent those of their affiliated organizations, or those of the publisher, the editors and the reviewers. Any product that may be evaluated in this article, or claim that may be made by its manufacturer, is not guaranteed or endorsed by the publisher.
